# Diagnostic performance of the noninvasive prenatal FetoGnost RhD assay for the prediction of the fetal RhD blood group status

**DOI:** 10.1007/s00404-021-06055-1

**Published:** 2021-04-09

**Authors:** Tobias J. Legler, Sandra Lührig, Irina Korschineck, Dieter Schwartz

**Affiliations:** 1 Department of Transfusion Medicine, University Medical Center Göttingen, Robert-Koch-Str. 40, 37075 Göttingen, Germany; 2 Ingenetix GmbH, Vienna, Austria; 3 Department of Blood Group Serology and Transfusion Medicine, Medical University of Vienna, Vienna, Austria

**Keywords:** NIPT-RhD, Diagnostic accuracy, Targeted anti-D prophylaxis, Multiple pregnancies

## Abstract

**Purpose:**

To evaluate the diagnostic accuracy of a commercially available test kit for noninvasive prenatal determination of the fetal RhD status (NIPT-RhD) with a focus on early gestation and multiple pregnancies.

**Methods:**

The FetoGnost RhD assay (Ingenetix, Vienna, Austria) is routinely applied for clinical decision making either in woman with anti-D alloimmunization or to target the application of routine antenatal anti-D prophylaxis (RAADP) to women with a RhD positive fetus. Based on existing data in the laboratory information system the newborn’s serological RhD status was compared with NIPT RhD results.

**Results:**

Since 2009 NIPT RhD was performed in 2968 pregnant women between weeks 5 + 6 and 40 + 0 of gestation (median 12 + 6) and conclusive results were obtained in 2888 (97.30%) cases. Diagnostic accuracy was calculated from those 2244 (77.70%) cases with the newborn’s serological RhD status reported. The sensitivity of the FetoGnost RhD assay was 99.93% (95% CI 99.61–99.99%) and the specificity was 99.61% (95% CI 98.86–99.87%). No false-positive or false-negative NIPT RhD result was observed in 203 multiple pregnancies.

**Conclusion:**

NIPT RhD results are reliable when obtained with FetoGnost RhD assay. Targeted routine anti-D-prophylaxis can start as early as 11 + 0 weeks of gestation in singleton and multiple pregnancies.

## Introduction

In alloimmunised pregnant women non-invasive prenatal testing (NIPT) can be applied to identify fetuses with increased risk due to the presence of at least one blood group specific nucleotide polymorphism (SNP) [1]. Since large-scale NIPT for RhD studies have been performed, the published confidence intervals for diagnostic sensitivity and specificity are relatively small. In contrast, smaller patient cohorts have been investigated to validate NIPT for other blood groups namely Kell, RhC, Rhc and RhE resulting in a very similar diagnostic accuracy compared with NIPT for RhD but with higher confidence interval [2–4]. To date, real-time PCR is the most common technology applied for the determination of fetal blood groups [1]. More recently, droplet digital PCR and next-generation sequencing have been proposed for noninvasive fetal molecular blood group genotyping, especially when antigens different from RhD have to be investigated [5–7]. Theoretically, compared with real-time PCR, the accuracy could be higher with these more modern methods. However, larger cohort studies still have to be done to provide evidence for this consideration.

It is very well established for over 50 years that the risk of Rhesus D (RhD) alloimmunisation and the number of subsequent cases of hemolytic disease of the fetus and newborn (HDFN) can be reduced by postpartal and routine antenatal anti-D prophylaxis (RAADP) [8–10]. However, a considerable number of anti-D-immunoglobulin doses are applied unnecessarily in pregnancies with RhD negative fetuses. Because of the scarce supply of anti-D immunoglobulin and possible adverse reactions there has always been the goal to restrict this treatment to women carrying RhD positive fetuses only (targeted RAADP).

Since the feasibility of targeted antenatal RAADP based on the result from a NIPT for the prediction of the fetal RhD status has been proposed by Dennis Lo’s group and a Dutch group in 1998 it took about 12 years until the first nationwide program for targeted RAADP was implemented in Denmark [11–13]. Until today many validation studies have been published and extensively reviewed which revealed an excellent diagnostic accuracy of NIPT for RhD [14–21]. It has to be stressed in the given context that only false-negative NIPT RhD results may have relevant consequences (i.e., increased risk for anti-D alloimmunisation). False-positive results will just lead to unnecessary anti-D immunoglobulin administration in such cases as without any NIPT RhD testing.

Despite extensive literature dealing with the diagnostic accuracy of NIPT assays already used in diagnostic laboratories for the prediction of the fetal RhD status, we identified the following three research questions which we address in this paper:

1. What is the diagnostic accuracy of a new commercially available real-time PCR assay?
2. Can targeted RAADP be applied as early as week 11 + 0 of gestation (wg)?
3. Is targeted RAADP applicable to multiple pregnancies?

## Methods

The FetoGnost RhD real-time PCR assay (Ingenetix GmbH, Vienna, Austria), not CE IVD approved yet, was evaluated by reviewing NIPT-RhD results and RhD blood group serology results from newborns from the Medical University of Vienna in a retrospective analysis. The study protocol was approved by the local ethical committee (approval no. 1927/2020). Oligonucleotides and probes for *RHD* exon 5 (VIC) and 7 (FAM) verified in a multicenter study were used with minor modifications [22, 23]. Exon 5 and 7 reagents were used in one multiplex PCR together with primers/probes for *RHD* exon 10 (NED) and a 76 bp synthetic oligonucleotide as internal positive control (IPC, Cy5). For real-time PCR instruments which lack the filter for Cy5, the IPC can also be tested with a VIC-labelled probe in a separate tube. The IPC target was added to the extraction tube to detect DNA extraction as well as amplification failures. IPC primer-binding sites were specific for mus musculus *ICAM1,* whereas the IPC probe binding site was specific for human *GAPDH*. Every sample was tested in triplicates resulting in a maximum of 9 *RHD* specific calls. Samples with 0–2 calls were interpreted as RhD negative, 7–9 calls as RhD positive, 3–6 calls as inconclusive.

In Vienna routine, NIPT-RhD has been performed since 2009 after *in-house* validation using 50 samples provided by the Göttingen group pursuant to guideline 98/79/EG of the European Commission for clinical decision making in all RhD negative pregnant women, either to advice the management in cases with anti-D alloimmunization or to apply targeted RAADP.

In general, all pregnancies at the Vienna Medical University’s Obstetrics department are considered to have some sort of risk, at least at some time during the course of pregnancy. Blood group data from all live births at this department have continuously been checked with NIPT RhD test results as NIPT RhD testing performance evaluation. Since a significant proportion of pregnancies were released from risk management and delivered in another hospital or pregnancies resulted in no live birth, there has been a relatively high number of NIPT RhD cases without a corresponding newborn’s RhD typing result in our lab (lost to follow-up).

All samples were collected locally, after informed consent was obtained, using 8 ml K2EDTA separator tubes (Ref 455021, Greiner Bio-One GmbH, Austria). Plasma was separated by centrifugation 10 min (4000 RCF) and the original tubes were stored at − 20 °C within 6 h after venipuncture until analysis. Cell-free fetal DNA was extracted manually using a spin column method (QIAamp DSP Virus Kit, Qiagen, Hilden, Germany) in the first years and since 2017 automatically (Maxwell, RSC ccfDNA Plasma Kit, Promega GmbH, Walldorf, Germany). Real-time PCR (StepOne Plus, Applied Biosystems, Foster City, CA, US) was performed according to the FetoGnost RhD handbook. In addition, an *SRY* sequence was detected as the fetal marker. In brief, primers RhD SRY-1F TGGCGATTAAGTCAAATTCGC (10 pmol/30 µl PCR), RhD SRY-72R CCCCCTAGTACCCTGACAATGTATT (10 pmol/30 µl PCR) and TaqMan® probe RhD SRY-30 T NED-CCTGACTGCTCTACTGC-MGB (RevComp, 5 pmol/30 µl PCR) were used to amplify part of the Y-chromosome in male fetuses. Furthermore, hypermethylated *RASSF1A* was analyzed as a second, sex-independent fetal marker according to the protocol previously described by Chan et al. [24]. One thermocycler profile was used for all real-time PCR assays. Following an incubation at 50 °C for 2 min and 95 °C for 20 s 60 2-step cycles with denaturation at 95 °C for 5 s and 60 °C for 1 min were performed.

Negative (aq. dest.) and positive (plasmid containing target sequences for *RHD*, *SRY*, *RASSF1A* and *ACTB*) controls have been included in each run. In addition, for each new lot of the assay a backup sample from a previous NIPT RhD testing with a confirmed follow-up of a RhD positive male child has been tested.

In general, the Vienna Immunohaematology laboratory has recommended the confirmation of negative NIPT RhD on a second sample (independent venipuncture) before any consequences (omission of anti-D immunoglobulin in non-immunized women, cessation of risk management in immunized women) are drawn. Because there was no single false-negative testing observed in the first 6 years of routine testing and of economic constraints, this recommendation was not stringently followed thereafter. One false-negative typing in a non-immunized woman that occurred in 2016 would most probably have been prevented if this strategy had been maintained. For this study, only the first testing results were included in the analysis.

Statistical analysis concerning calculation of confidence interval (CI, Wilson interval) using R (version 4.0.2 (2020-06-22) was performed in collaboration with the Department of Medical Statistics (University Medical Center Göttingen).

## Results

**Fig. 1:**
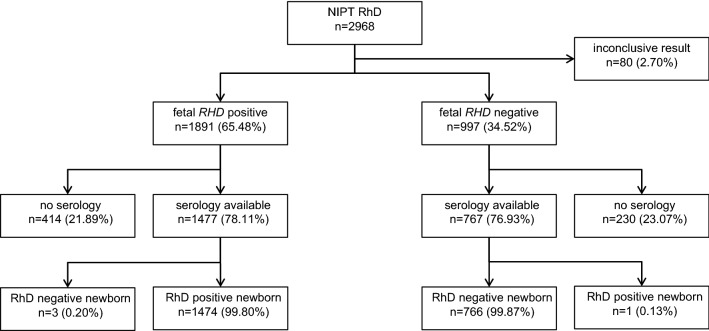
Flow of participants. NIPT RhD was performed with the FetoGnost RhD assay taking fetal markers *SRY* and hypermethylate *RASSF1A* into account. The results of the newborn’s RhD status serologically determined from cord blood were taken as a reference

The diagnositic accuracy of the FetoGnost RhD assay was determined from the continuous performance evaluation of routine testing. NIPT-RhD together with controls for hypermethylated *RASSF1A* as well as *SRY* was performed in 2968 pregnancies at week 5 + 6 until week 40 + 0 of gestation (median 12 + 6 wg). About half of the samples (50.9%) were collected in the first trimester. Patients were between 16 and 50 years (median 32 years). Conclusive results were obtained in 2888 (97.30%) cases (Fig. 1). Because rather low ct-values were observed and a maternal *RHD* positive genotype was suspected (*n* = 15), or the number of *RHD* specific calls were not conclusive (*n* = 29) or 1–2 exons did not show any positive result and a D-variant in the fetus was suspected (*n* = 9), or the *RHD* result was negative, but the fetal controls were negative or inconclusive (*n* = 27), no conclusive NIPT RhD results was reported in 80 (2.70%) cases. Among these 27 NIPT RhD negative cases with inconclusive fetal markers, there was one at 11 + 3 wg with 7 of 9 negative reactions in the FetoGnost RhD test, where the fetus was tested RhD positive after birth, 21 newborns were RhD negative and in 5 cases no result from a newborn was available.

In 2244 of 2888 (77.70%) cases with conclusive NIPT RhD result, information about the RhD phenotype of the newborns was available from the immunohematology laboratory. If at least one child was RhD positive or weak D in multiple pregnancies, this was counted as “one RhD positive newborn” in Fig. 1 and for the calculation of the sensitivity. If all children in multiple pregnancies were RhD negative in multiple pregnancies, this was counted as “one RhD negative newborn” in Fig. 1 and for the calculation of the specificity. 1475 women delivered at least one RhD positive (*n* = 1462) or weak D child (*n* = 13) of which 1474 serological RhD results were predicted correctly, the sensitivity was 99.93% (95% CI 99.61–99.99%). A frozen backup sample of the single false-negative case, a singleton pregnancy at 13 + 4 wg, tested *RHD* positive in a repeat test. In 769 cases, one or more RhD negative children were born. In three of these cases, NIPT RhD predicted an RhD positive newborn. The specificity of the FetoGnost RhD test was 99.61% (95% CI 98.86–99.87%) and the accuracy 99.82% (95%CI 99.54–99.93%). Even when results from samples collected beyond 19 wg were excluded (no errors in this cohort, *n* = 488), a high sensitivity of 99.91% (95% CI 99.50–99.98%, *n* = 1144), specificity of 99.51% (95% CI 98.57–99.83%, *n* = 612) and accuracy of 99.77% (95%CI 99.42–99.91, *n* = 1756) was observed.

During the observation period, NIPT RhD was performed in 199 pregnancies with twins and in 7 pregnancies with triples. These cases are included in the numbers of Fig. 1 and in the calculation of sensitivity and specificity. In 205 (99.5%) of these 206 cases, a conclusive NIPT RhD result was obtained and in 203 cases the phenotype of the newborns was reported. Complete concordance between NIPT RhD result and cord blood results was observed in 196 pregnancies with twins and in 7 pregnancies with triplets, respectively. In 141 cases, at least one child was RhD positive, in one case, both twins were weak D, and in 61 cases, all children were RhD negative.

## Discussion

Recently, data from about 60.000 study participants were pooled in a large meta-analysis for the purpose to determine the diagnostic accuracy of NIPT RhD [20]. However, in this systematic review, only studies were included, which evaluated lab developed (*in-house*) tests. In contrast, the literature dealing with the validation of commercial NIPT RhD test kits is less comprehensive. The SensiGene® RHD assay (Sequenom, San Diego, CA, USA) detects exons 4, 5 and 7 of the *RHD* gene, *RHD* psi and three sequences on the Y chromosome (SRY, TTTY, DBY) for the control of fetal DNA in male fetuses. Initially, Bombard et al. described a sensitivity of 97.2%, and a specificity of 96.8% (*n* = 207) of the SensiGene® RHD assay taking the newborn’s serological RhD result as a reference. However, in comparison with genotype reference, they observed an increased sensitivity (100.0%) and specificity 98.3% (*n* = 199) [25]. In a subsequent observational study, Moise et al. found one false-negative result in 324 RhD positive fetuses due to mislabeling of a collection tube and 2 false-positive results in 136 RhD negative fetuses when testing between 11 and 29 wg [26].

Protected by an exclusive patent license, the Free DNA Fetal Kit® RhD (Institut de Biotechnologies Jacques Boy, Reims, France) was until recently the only real-time NIPT RhD PCR assay commercially available in Europe. In a validation study performed to receive CE-approval of this test kit, Roullica-Le Sciellour et al. described a sensitivity of 100% and a specificity of > 99% (two false-positive) in 300 plasma specimen tested between 10 and 34 weeks of gestation [27]. In addition, Londero et al. found more recently a complete concordance between the Free DNA Fetal Kit® RhD result obtained from week 11 + 6 of gestation until term and RhD phenotype at birth in all 133 cases investigated [28]. In comparison with these studies, the FetoGnost RhD assay was evaluated with more pregnant women during this continuous performance evaluation in Vienna. Diagnostic accuracy of this test kit was as high as the diagnostic accuracy determined in larger nation-wide screening studies with *in-house* NIPT for RhD [14, 29, 30]. One false-positive result could be explained by a maternal non-coding *RHD* variant, in a second case, two fetuses vanished during a pregnancy with triplets and only an RhD negative fetus survived and for the third false-positive result no conclusive reason was found. In this case and in one false-negative case with a positive repeat test, either the first test was wrong due to a technical failure or a wrong sample was tested as a consequence of human error.

Beyond RAADP, anti-D administration is also required earlier in RhD negative pregnancy whenever there are clinical signs or risks for fetomaternal hemorrhage [8]. Therefore, we were especially interested in the sensitivity and specificity of the FetoGnost assay in the first trimester. Wikman and co-workers described in 4118 pregnancies an increase of sensitivity of a single-exon fetal *RHD* assay during the course of pregnancy. After exclusion of samples analyzed before 10 wg, the sensitivity was 99.3% and it increased up to 100% when results from 22 wg or later were included into the calculation [30]. In a large study performed in seven maternity units in England, Chitty et al. described a sensitivity of 99.83%, 99.67%, 99.82% and 100% at 11–13, 14–17, 18–23 and > 23 completed wg, respectively. Although false-negative results were rare, they were mainly observed earlier in gestation [31]. In contrast, another study performed in 10–14 wg did not observe false-negative results in 416 serum samples (2.2% inconclusive). However, the specificity of an assay based on replicate testing of *RHD* exon 10 with 95.2% was lower than in the previous studies [32]. In our performance evaluation study, the sensitivity of 99.93% and specificity of 99.61% were as high as sensitivities and specificities determined in the second trimester with large-scale screening studies [14, 21, 29, 33]. Notably, our single false-negative result was observed at the beginning of the second trimester.

In multiple pregnancies, no false-negative NIPT RhD results were described in 3 studies with overall 92 cases [30, 34, 35]. With this publication, we add another 203 cases, where NIPT for RhD correctly predicted the risk of anti-D alloimmunization if that at least one newborn was RhD positive or weak D, whereas no false-positive genotyping results were reported in 61 cases. Therefore, targeted RAADP is reasonable also in multiple pregnancies.

In a recent review Yang et al. concluded, that false-negative results are rather rare after 13 wg. [18]. Due to the false-negative case at 13 + 4 wg in our diagnostic accuracy study we support the conclusion of the German Association for Transfusion Medicine and Immunohematology (DGTI) that a single NIPT for RhD test beyond 19 wg is a safe procedure to apply targeted RAADP in the second trimester [36]. If testing for fetal markers was not been performed in Vienna, one more false-negative case would have occurred at 11 + 3 wg. Therefore, we suggest, that before 20 wg a control for fetal DNA should confirm the presence of cff DNA if NIPT for RhD predicts an RhD negative fetus and if no second sample for confirmation is available due to public health economic considerations. The suitability of testing hypermethylated *RASSF1A* with real-time PCR as fetal marker has also been demonstrated by other groups [37–39].

In conclusion, NIPT RhD performed with the FetoGnost RhD assay delivers reliable results in the first and second trimester both in singleton and multiple pregnancies, respectively. However, even if an analytical process is 100% reliable through a maximum of automation, human errors during blood collection or labelling of blood tubes never can be completely excluded. Based on the analysis of underlying risks for the individual patient, a single NIPT for RhD can be considered sufficient for targeted anti-D prophylaxis. In contrast, if the management of an alloimmunized woman with antibodies against paternally inherited blood group antigens has to be stratified, a negative NIPT for RhD has to be confirmed from a second, independent blood drawing, because errors due to mislabeling of blood tubes can never be excluded.

## Data Availability

All authors made sure that all data and materials support the published claims of this manuscript and comply with field standards.
